# Current Status of Matrix-Assisted Laser Desorption/Ionization–Time-of-Flight Mass Spectrometry (MALDI-TOF MS) in Clinical Diagnostic Microbiology

**DOI:** 10.3390/molecules25204775

**Published:** 2020-10-17

**Authors:** Sachio Tsuchida, Hiroshi Umemura, Tomohiro Nakayama

**Affiliations:** Divisions of Laboratory Medicine, Department of Pathology and Microbiology, Nihon University School of Medicine, Tokyo 173-8610, Japan; tsuchida.sachio@nihon-u.ac.jp (S.T.); nakayama.tomohiro@nihon-u.ac.jp (T.N.)

**Keywords:** bacterial identification, blood culture, clinical proteomics, matrix-assisted laser desorption/ionization–time-of-flight mass spectrometry (MALDI-TOF MS), sample preparation

## Abstract

Mass spectrometry (MS), a core technology for proteomics and metabolomics, is currently being developed for clinical applications. The identification of microorganisms in clinical samples using matrix-assisted laser desorption/ionization–time-of-flight mass spectrometry (MALDI-TOF MS) is a representative MS-based proteomics application that is relevant to daily clinical practice. This technology has the advantages of convenience, speed, and accuracy when compared with conventional biochemical methods. MALDI-TOF MS can shorten the time used for microbial identification by about 1 day in routine workflows. Sample preparation from microbial colonies has been improved, increasing the accuracy and speed of identification. MALDI-TOF MS is also used for testing blood, cerebrospinal fluid, and urine, because it can directly identify the microorganisms in these liquid samples without prior culture or subculture. Thus, MALDI-TOF MS has the potential to improve patient prognosis and decrease the length of hospitalization and is therefore currently considered an essential tool in clinical microbiology. Furthermore, MALDI-TOF MS is currently being combined with other technologies, such as flow cytometry, to expand the scope of clinical applications.

## 1. Introduction

Mass spectrometry (MS) applications have expanded significantly in recent years [1]. After years of efforts by concerned organizations and academic societies, MS was officially approved by the Japanese Ministry of Health, Labor and Welfare as a medical device in 2017. Specifically, the pharmaceutical safety and environmental health bureau notification (No. 0311–1) of the pharmaceutical affairs law has been enacted to secure the quality, efficacy, and safety of products, including pharmaceuticals and medical devices.

MS has shown much progress within two main categories: searching for novel diagnostic biomarkers via comprehensive proteome/metabolome analyses [2,3,4] and clinical microbiology testing [5,6,7,8,9,10,11,12,13]. The present review focuses on clinical microbiology testing. 

Matrix-assisted laser desorption/ionization–time-of-flight mass spectrometry (MALDI-TOF MS) is one of the most widely used MS methods applied to the identification of microorganisms in clinical samples [5,6,7,8,9,10,11,12,13]. This new application has proven useful in the diagnosis and treatment of infectious diseases that require a swift response. Commercially available devices include those with integrated analytical capabilities, such as the MALDI Biotyper (Bruker Daltonics, Bremen, Germany) and VITEK^®^ MS (bioMérieux, Marcy l’Etoile, France). Tests that are carried out using these devices can be reimbursed via health insurance in Japan.

Identifying microorganisms using MALDI-TOF MS is convenient, rapid, and accurate while also being cost-effective. Thus, this method has revolutionized microbial identification (ID) in clinical microbiology laboratories [14,15]. Initially, this approach was applied to bacterial colonies grown on agar plates. However, expanding the applicability to direct analysis of clinical specimens without the need for prior culture or subculture has increased the usefulness of this technology. 

In this review, we describe MALDI-TOF MS-based microbial ID, with particular emphasis on the methods developed so far to identify microorganisms. We highlight the increased use of MALDI-TOF MS to identify microorganisms, as well as its use in combination with other methods.

## 2. Historical Transition

The development of microbial ID using MS [16] began in the 1970s when gas chromatography–mass spectrometry (GC-MS) was used to identify microorganisms [17,18]. The fingerprinting technique using pyrolysis MS dates back to the 1950s [19]. Moreover, multiple studies on the use of MS peak patterns to analyze bacterial fatty acids attracted considerable attention [20]. 

In 1988, Tanaka et al. developed soft laser desorption. This technology involves ionization of proteins without destroying them, thus opening up for major advances and opportunities in the field [21]. With soft laser desorption significantly contributing to the development of the MALDI method, Tanaka was later granted the Nobel Prize in Chemistry. The MALDI method was combined with time-of-flight mass spectrometry (TOF MS) to become MALDI-TOF MS, which quickly became a widely used technique. In 1994, Cain et al. [22] demonstrated that MALDI-TOF MS protein profiling using α-cyano-4-hydroxycinnamic acid as a matrix material was useful for rapid bacterial ID. However, at that time, pretreated bacteria were used. Due to additional innovation, bacterial colonies can nowadays be directly applied to an MS plate without any special pretreatment prior to MS analysis [23,24].

Since 2009, numerous clinical studies have been published involving the use of MALDI-TOF MS equipment, which is now viewed as mandatory for bacterial testing laboratories in hospitals.

## 3. MALDI-TOF MS-Based Identification Methods in Clinical Practice

In daily practice, clinical specimens, such as sputum, pus, swab sticks, and urine from patients, are first developed on medium, and microorganisms are isolated as colonies. The pathogens are evaluated based on observations of the cultured colonies, the patient’s clinical information, microscopy using Gram staining, and the number of microorganisms. The ID of the microorganisms is then obtained using a MALDI-TOF MS procedure.

**Figure 1:**
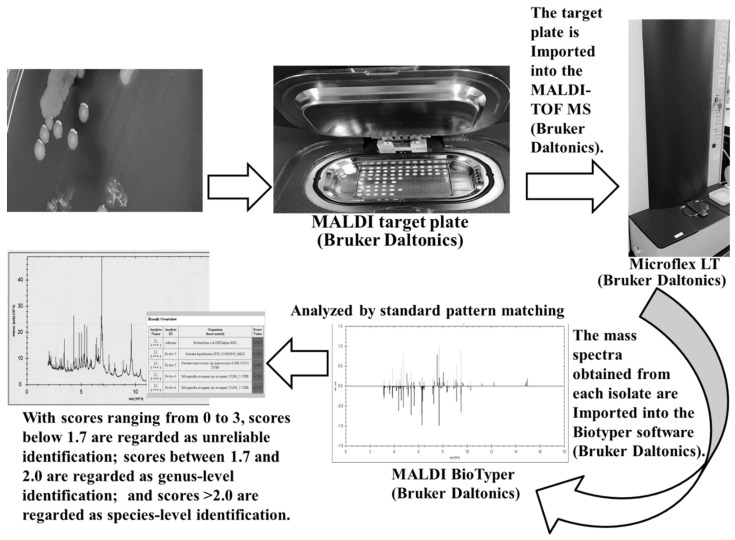
Identification process using matrix-assisted laser desorption/ionization–time-of-flight mass spectrometry (MALDI-TOF MS) (MALDI Biotyper). Colony scraping technique using a sterile toothpick. Thecolony adhering to the toothpick was directly transferred onto a MALDI target plate for spotting. Immediately, 1 µL of 70% formic acid and matrix were spotted onto the MALDI target plate, and bacterial ID was performed with MALDI-TOF MS analysis using a MALDI Biotyper.

Figure 1 shows the ID process using MALDI-TOF MS (MALDI Biotyper). The process can be summarized as follows: (1) a colony is transferred to the sample plate, which will later be exposed to laser irradiation. When the matrix has been added and dried, the plate is inserted into the MS. (2) The plate is then irradiated using the laser, and the proteins in the bacteria (primarily ribosomal proteins) are ionized; the time required for these ionized proteins to fly to the detector determines the mass-to-charge ratio (*m*/*z*) of the component proteins, and the intensity of the signal provides the mass spectrum (pattern). (3) The mass spectrum obtained is then compared with those in a reference database to identify the bacteria. The primary matrix used for bacterial ID is α-cyano-4-hydroxycinnamic acid (CHCA). CHCA works by (1) efficiently absorbing the energy of the laser, (2) promoting the ionization of the sample by providing protons, and (3) preventing the sample from breaking down. MALDI-TOF MS can shorten the ID process by about 1 day. Thus, MALDI-TOF MS enables ID of microorganisms at an earlier stage, which again may result in quicker clinical response, including earlier intervention with empirical therapy [25,26,27,28]. Using a new colony for diagnostic testing increases the chances of accurate ID. For fast-growing bacteria, ID is possible on the first day after culturing or empirically within 2–3 days. For slow-growing bacteria, the ID time is based on the growth rate. 

In a study using *Escherichia coli,* Ryzhov V et al. reported that approximately half of the MALDI mass peaks detected in the range of 4000–20,000 Da were ribosomal proteins (including some with post-translational modifications), and the other peaks represented DNA-binding proteins and cold-shock proteins [29]. The mass spectral pattern is unique to the sample organism, but it has several common peaks depending on the bacterial species, and these peaks are used to identify the organism. Main spectra profiles (MSPs), which are schematized mass spectral patterns of thousands of known standard strains, are registered in a database, and the MSPs of mass spectral patterns of sample strains are compared with those of known standard strains to enable bacterial identification by selecting the most similar ones and using them to distinguish different species and genera. To ensure validity of the MALDI tests, isolates were clearly identified using MALDI–TOF MS; these identifications were shown to be consistent with the results of 16S rRNA sequencing. Furthermore, positive blood culture isolates were subjected to in-parallel direct identification by MS fingerprinting and conventional subculturing for reference identification.

The primary recommended method for obtaining bacterial IDs from colonies is the cell smear (direct smear) method. This method involves careful selection of a colony from the medium where it was grown and thinly applying it to the MS sample plate and then adding the matrix on top of it and allowing it to dry, followed by taking measurements. Using this smear method, intestinal bacteria and other Gram-negative, rod-shaped bacteria can be identified with high precision; however, the probability of correctly identifying Gram-positive bacteria, such as staphylococci and enterococci, or yeasts is still somewhat limited. This is possibly due to differences in the cell wall structure; poor extraction of proteins from microbial cells usually results in insufficient spectral information required for accurate ID. In such cases, to promote cell wall destruction after applying the colony to the sample plate, the formic acid extraction (on-plate) method is applied to increase the probability of accurate identification [30,31,32,33,34]. Hence, tests may first be performed using the cell smear method; if the ID score is insufficient, then the on-plate method is used. Failure to obtain a sufficient mass spectrum using the on-plate method may be attributed to a low amount of protein being extracted from the sample. In this case, the ethanol–formic acid extraction method is effective. This method relies on a combination of ethanol treatment followed by protein extraction with formic acid and acetonitrile. Furthermore, in certain cases, beads may be used to ensure physical destruction of the cell walls. As an example, we previously developed a new extraction method for *Nocardia*, with ethanol–formic acid extraction and silica beads added to the basic methodology [35]. Silica beads can physically shake and break up even the strong spores and cell walls of gram-positive bacteria more efficiently, thereby improving the extracting bacterial proteins. Furthermore, silica beads are an effective tool for extracting bacterial proteins from acid-fast bacteria [36].

Two systems and their associated databases, the MALDI Biotyper (Bruker Daltonics) and the VITEK^®^ MS (bioMérieux), are currently widely used for MALDI-TOF MS-based bacterial ID [37,38,39,40,41,42]. Although the analytical principles of the two systems are similar, they differ with respect to the databases used and the construction of the diagnostic algorithms. Additionally, the modes of data presentation differ to some extent. For example, in the MALDI Biotyper system, the results of the pattern-matching process are presented as proposed by the manufacturer, with scores ranging from 0 to 3. Scores < 1.7 are regarded as unreliable, scores between 1.7 and 2.0 can be considered genus-level IDs, and scores > 2.0 can be used to provide ID at the species level [39]. Conversely, the Vitex^®^ MS system generates a confidence score, which is presented as a percent probability [40]. The percentage probabilities for a correct ID range from 60 to 99, with values closer to 99 indicating a closer match. Organisms for which percent probabilities < 60 are obtained should be considered unidentified [40].

MALDI–TOF mass spectrometry was successfully attuned for identifying fungi. This method is chiefly used for the routine identification of yeast; conversely, further development is necessary, most notably in sample preparation protocols and database libraries, to use this identification approach for other groups of fungi (such as dermatophytes and filamentous fungi). The misidentification or non-identification of fungal genera and species by MALDI–TOF mass spectrometry is essentially caused by mistakes, absences, or incomplete reference spectra in databases. The species-level identification of many fungi by the same method remains somewhat limited. One reason why it is difficult to use the MALDI–TOF mass spectrometry microbial identification method for fungi is that fungal species, mainly filamentous fungi that are lacking protocols to provide sufficient identification results at the species level, cannot be identified. Further study of pretreatment methods will be necessary to obtain stable identification results similar to those of common bacteria. Only results with concordance scores above the manufacturer’s proposed cutoff for reliable species-level identification (2.0) were retained. If no corresponding species-level identification could be obtained from the biochemical typing and mass spectrometry fingerprinting, full-length 16S rRNA gene sequences were acquired [43]. 16S rRNA gene analyses were used for species-level delineation based on assigning the closest type strain.

## 4. Direct Identification from a Positive Blood Culture Bottle

Sepsis is a severe infection that requires rapid diagnosis and treatment. In situations where it is unclear which particular organs are affected, blood cultures are useful for obtaining information concerning the infecting organism. The use of MALDI-TOF MS for identifying bacteria requires a bacterial abundance above a certain threshold; therefore, this method is often used after confirming colony formation. However, in cases where speed is particularly essential, it is possible to perform MS-based ID directly using a culture solution as soon as the blood culture is considered positive. This is one of the most promising technologies currently available for the ID of microbial pathogens directly from positive blood culture bottles [44,45,46,47,48] and has considerable significance as a rapid diagnostic method, reaching a positive predictive value of 60–80% for different species of microorganisms [49]. Differentiating microorganisms from host cells is a critical step for successful ID, and several laboratory-developed and commercially available protocols have been reported for this purpose, as reviewed elsewhere [50,51,52]. The following are representative examples of laboratory-developed test protocols: (1) stepwise sedimentation of blood cells and microorganisms [51]; (2) low-speed centrifugation for removing blood cells, followed by an additional lysis procedure [52]; (3) removal of blood cells using serum separator tubes [53]; and (4) saponin use [54]. Meanwhile, to the best of our knowledge, only three commercial protocols are currently available: the Sepsityper^®^ kit (Bruker Daltonics) [55], the VITEK^®^ MS blood culture kit (bioMérieux) [56], and the rapid BACpro^®^ II kit (Nittobo Medical Co., Tokyo, Japan) [57,58].

Quick bacterial ID by MALDI-TOF MS on blood culture material enables rapid administration of relevant treatment to the patient, resulting in decreases in the time spent in intensive care units and length of hospitalization [59,60,61]. 

In clinical samples, multiple proteins may be present in high quantities that do not come from bacteria, such as hemoglobin in blood cultures; therefore, pretreatment steps, such as separation of blood cells, are required to recover the bacterial cells selectively [52]. Rapid ID of bacteria using MALDI-TOF MS has already been integrated as a routine test in many institutions; however, no standardized clinical pretreatment method is available, so each institution appears to use its own methods. Although bacterial ID via MS is an efficient method to rapidly identify bacterial genera and species, most pretreatment methods published to date require multiple high-speed centrifugation steps, resulting in lengthy processing times. Thus, making the pretreatment process quicker and more convenient is ideal in this respect.

Pretreatment of samples using MS methods is necessary for direct identification of microorganisms from blood cultures. We previously investigated the functionality of various methods and kits [57,62,63]. Among these, the MALDI Sepsityper^®^ kit (Bruker Daltonics) is extensively used [64,65]. This involves mixing 200 μL of kit lysis buffer with 1 mL of blood culture to break down the blood components of the sample; the bacterial cells are subsequently harvested via high-speed centrifugation. Another method involves breaking down blood components in 2 mL of blood culture using 1 mL of lysis buffer and then collecting the bacteria using a 0.45-µm Millipore Express PLUS with a 47 mM-diameter membrane filter. The VITEK^®^ MS blood culture kit (bioMérieux) collects bacteria using a filter. The bacteria-collecting apparatus uses a glass manifold and a vacuum pump. After the bacteria are collected, the on-plate method is used for extraction. By using bespoke methods, the proteins from red blood cells and other host cells in the blood culture are selectively removed, and the bacteria can be collected efficiently. Moreover, we recently contributed to the development of a method, which selectively removes hemoglobin, an inhibiting factor, and uses a copolymer to collect the bacteria [57]. In this method, the negative charge on the surface of the microorganisms is used to capture the microorganisms via attraction to the positive charge of polyallylamine (cationic granules). This product has been improved and turned into a practical pretreatment kit produced in Japan. It is sold in Japan and overseas as rapid BACpro^®^ II and is currently undergoing clinical evaluation [58,64]. 

Moreover, rapid bacterial ID from positive blood cultures has progressed to the genetic level [65]. An automated, simultaneous multiphase gene detection system, which detects pathogens and drug resistance genes, has been described in the literature [66]. However, these methods are still in the developmental stage. Therefore, in the future, MALDI-TOF MS assays should be compared with gene detection methods in terms of rapidness and accuracy.

The ability of MALDI-TOF MS to correctly identify all microbial species involved when multiple species contribute to the pathology is a topic for future research. To summarize, advances in the use of MALDI-TOF MS for direct ID of microbes from blood culture samples are needed.

## 5. Direct Identification of Microbes in Urine or Cerebrospinal Fluid (CSF)

Currently, urine tests such as dipsticks are important first-line diagnostic tools. These tests detect three key groups of early symptoms, namely, diseases of the kidney and the urinary tract, carbohydrate metabolism disorders (typically diabetes mellitus), and liver diseases and hemolytic disorders. If the quantity of bacteria in a urine sample is at or above a certain level, the sample can be directly used for bacterial ID for diagnosing urinary tract infections (UTI) [67]. Notably, UTI are the most frequent type of bacterial infection in humans. In a study of 220 urine samples in which monomicrobial bacterial growth was higher than 105 CFU/mL, the organism could be identified to the species level in 202 of the samples (91.8%) [68]. Veron et al. compared three ID methods (differential centrifugation, urine filtration, and 5 h bacterial cultivation on solid culture media) based on their ability to identify bacteria and their potential as a routine tool for microbiology laboratories [69]. A higher proportion of correct MALDI-TOF MS bacterial ID was obtained through filtration (78.9%) and the culture-based method (84.2%) compared with centrifugation (68.4%) [69]. These results demonstrated that a short culture step is a straightforward and efficient sample preparation method, enabling fast and reliable ID of uropathogens by MALDI-TOF MS.

Meningitis involves inflammation of the meninges, and bacterial meningitis in particular is a critical condition, requiring rapid intervention. Currently, the first step in detecting the bacterial pathogens responsible for meningitis is microscopy of Gram-stained smears of CSF to determine whether bacteria are present. Patients with positive smears receive treatment with broad-spectrum antibiotics based on the Gram staining results. To date, only a limited number of studies have described the use of MALDI-TOF MS for the direct detection of microorganisms causing bacterial meningitis. 

Direct ID of bacteria in the CSF enables rapid bacteriological diagnosis and early appropriate treatment, which is crucial in bacterial meningitis. Because multiple cell types may exist in CSF samples, the nonrelevant cells (typically host cells) must be removed from the sample before analysis. A case was recently published, in which analysis of a CSF sample by a MALDI Biotyper led to a rapid diagnosis of bacterial meningitis caused by *Klebsiella pneumoniae* [70].

These results suggest that the direct ID of bacteria at or above a certain quantity in urine or CSF samples is possible. However, similar to the situation for blood cultures, further studies should be performed to address the current limitations in the detection and differentiation of >1 bacterial species involved in such infections.

Christner et al. required a minimum of 10^6^ CFU/mL of bacteria to reliably identify bacteria in blood culture bottles with MALDI–TOF mass spectrometry [71]. A comparison of reports of several urine samples from bacterial cases shows a sensitivity of approximately 10^4–6^ CFU/mL [67,69,72]. Therefore, it is presumed that a similar amount of CSF sample would be required. Moreover, MALDI–TOF mass spectrometry identification required a greater minimum concentration of bacteria than that required for the 16S rRNA gene analyses. 

## 6. Application of MALDI-TOF MS to Veterinary Medicine

MALDI-TOF MS has been applied in the field of veterinary medicine as well. Wilson et al. cultured milk samples submitted from a commercial dairy farm from recently calved cows or clinical mastitis cases and identified 181 isolates by conventional biochemical testing, MALDI-TOF MS, and 16S ribosomal DNA sequencing analysis. These three methods showed high concordance, and all of them were considered as valuable for identifying bacteria isolated from dairy cow milk [73]. 

## 7. MALDI-TOF MS Combined with Other Methods

Recently, the combination of MALDI-TOF MS with other methods has been reported for the ID of bacteria. Flow cytometry (FCM), a widely applied laser-based technology primarily used to measure fluorescence intensity, enables comprehensive analysis of the immune system and allows for the ID of various cell populations and deep analysis of the immune response elicited by novel therapeutics [74,75,76]. Gu et al. used a separation gel adsorption method and then applied MALDI-TOF MS combined with FCM to quickly identify bacterial pathogens isolated from a positive blood culture and performed a drug susceptibility test. This method was able to identify 71%, 74%, and 88% of Gram-negative bacteria, Gram-positive bacteria, and fungi to the species level, respectively. The results obtained using MALDI-TOF MS to identify pathogens agree with the results obtained using VITEK^®^2 (bioMérieux); both methods can identify 90% of bacteria to the species level. The method by Gu et al. can identify 75% of fungi to the species level, which is superior to the VITEK^®^2 assay, which is able to identify 60% of the tested fungi to the species level [77].

Attempts have been made to use MALDI-TOF MS to estimate antimicrobial susceptibility. The MALDI Biotyper antibiotic susceptibility test rapid assay (MBT ASTRA) is a novel semiquantitative method for susceptibility testing of microorganisms, including fungi. This method is a phenotypic assay, comparing microbial growth after incubation in the absence or presence of different concentrations of antimicrobial agents. Cell growth is estimated from a comparison of areas under the curve of the MALDI-TOF MS spectra of different incubation conditions [78,79,80].

Another area of significance for employing MALDI-TOF mass spectrometry is the detection of antibiotic resistance associated with identified bacteria, which has been extensively studied elsewhere; however, further research must be conducted on the application of MALDI–TOF mass spectrometry to detect antibiotic resistance on a routine basis. Spectra were automatically interpreted by Compass software (4.1) with a licensed MBT Subtyping Module. The software automatically identifies respective peaks (PSM-mec and δ-toxin) based on pre-processing of the spectra and determination of significant peaks. The detection of MRSA using MALDI–TOF mass spectrometry mediated by the peptide and phenol-soluble modulin (PSM-mec) linked to the class A mec gene complex present in SCCmec cassettes types IIIII and VIII of MRSA strains has been commercially available [81].

The automation of colony picking for matrix-assisted laser desorption/ionization time-of-flight mass spectrometry (MALDI–TOF MS) identification and antibiotic susceptibility testing is still under development. Chudejova et al. compared the results of average identification scores for routinely used manual deposition (semi-extraction for yeasts) with the results of automatic deposition using a MALDI Colonyst robot [82]. Spotting by MALDI Colonyst significantly increased the identification score of bacteria compared with the routine diagnostic process.

## 8. Application of Liquid Chromatography–Mass Spectrometry for Microbial ID

In addition to the increased practical application of MALDI-TOF MS, the combination of electrospray ionization (ESI) and liquid chromatography–mass spectrometry (LC-MS/MS) is becoming popular as a rapid quantitative technique for low molecular weight compounds. LC-MS/MS is an essential tool for analyzing medicinal toxicants and therapeutic drug monitoring in forensic medicine [83,84]. The extent of the scientific literature relating to bacterial ID using LC-MS/MS has also gradually increased [85,86,87,88]. A method for the simultaneous ID of staphylococci and the detection of staphylococcal antimicrobial susceptibility also has been reported, although this method is currently still in development [89]. While rapid ID of microorganisms by MALDI-TOF MS is becoming the mainstay of testing in clinical practice, the role of LC-MS/MS in this area has the potential to increase.

## 9. Conclusions

In the present article, we reviewed the advantages of MALDI-TOF MS in clinical microbiology and various technological innovations that have been developed for sample preparation in the ID of bacteria. The applications of MS in the field of clinical microbiology are not just limited to MALDI-TOF MS. LC-MS/MS or MALDI-TOF MS combined with other technologies has also begun to be applied. With the advances in this field, MS technology may one day be used more commonly than now for microbial typing studies and the detection of resistant bacteria. Currently, although the use of MS methods in the clinical ID of microorganisms is possible, traditional bacteriological methods such as the quality control of clinical samples (such as sputum), Gram staining results, and colony growth observations remain important. Furthermore, regardless of how analytical methods including MS progress, these methods must be evaluated and implemented by experienced clinical microbiologists parallel to the use of conventional techniques. In view of this, the development of excellent human resources is essential along with improvements in the application of novel clinical laboratory methods [6,90].
